# Targeted assembly recovers high ammonia monooxygenase diversity in mudflat intertides

**DOI:** 10.1128/msystems.00620-25

**Published:** 2025-09-24

**Authors:** Mengqi Wang, Wen Song, Jiayin Zhou, Mengzhi Ji, Kai Ma, Yan Li, Qichao Tu

**Affiliations:** 1 Institute of Marine Science and Technology, Shandong University, Qingdao, Shandong, China; 2 Shandong Key Laboratory of Intelligent Marine Engineering Geology, Environment and Equipment, Qingdao, Shandong, China; 3 Southern Marine Science and Engineering Guangdong Laboratory, Zhuhai, Guangdong, China; Pacific Northwest National Laboratory, Richland, Washington, USA

**Keywords:** shotgun metagenomes, targeted assembly, ammonia monooxygenase, genetic diversity, *amo *operons, mudflat intertidal zones

## Abstract

Microbial communities in the Earth’s biosphere mediate various biogeochemical cycling processes that are essential to maintain ecosystem multi-functioning and stability via the functional genes they carry. Although progress is being made, obtaining high-quality sequence data sets for microbial functional genes in complex environments remains challenging, both technically and in terms of computational resources required. In this study, using the *amo* gene family encoding ammonia monooxygenase as an example, we aimed to recover important microbial functional genes from shotgun metagenomes via targeted assembly. Comparing to conventional assembly approaches such as single-sample and multi-sample assembly, targeted assembly recovered much higher *amo* gene diversity while requiring substantially less computational resource and shorter running time. In addition, *amo* genes recovered by targeted assembly were found with fewer chimeras. Meanwhile, more *amo* operons were recovered. Not only were the commonly known *amoABC* subunits observed, but also the less commonly found subunits, like *amoX* and *amoE*. Notably, the archaeal *amoA* subunits recovered by targeted assembly represented the most “super-clades” for ammonia monooxygenase, including NT-α, NT-γ, NP-γ, NP-ζ, and NP-η, demonstrating the advantage of targeted assembly over conventional approaches. Comparable spatial patterns, such as taxa-area and distance-decay relationships, were also observed for the recovered *amo* assemblages. This study demonstrated an efficient route to recover microbial functional genes from shotgun metagenomes with minimal computational resource and running time.

**IMPORTANCE:**

Microbial communities play critical roles in the Earth’s biosphere by mediating various biogeochemical cycles of essential elements and maintaining ecosystem stability and multi-functioning through the functional genes they carry. However, recovering the key functional genes from such complex communities remains challenging. Both advantages and limitations exist for different technologies. In this study, using the *amo* gene family as an example, we show that targeted assembly enables accurate and rapid recovery of high-quality *amo* sequences from shotgun metagenomes, consuming minimal computational resources and running time. Compared to conventional full-assembly approaches, the *amo* sequences recovered by targeted assembly are found with more operons, higher (phylo)genetic diversity, and fewer chimeras. This study provides an efficient alternative route for recovering microbial functional genes, particularly when computational resources are limited.

## INTRODUCTION

Microorganisms play critical roles in the Earth’s biosphere by mediating various biogeochemical cycles of essential elements and maintaining ecosystem stability (1–3). One such example is the nitrogen cycling (4–7), through which different forms of nitrogen (e.g., ammonium, nitrate, nitrite, nitric oxide, nitrous oxide, and nitrogen gas) are transformed and cycled across Earth’s spheres. The conversions of different nitrogen forms between different redox states are carried out by the enzymes/proteins encoded by corresponding microbial functional genes. For instance, ammonia monooxygenase is a key nitrogen-transforming enzyme encoded by the *amo* gene, which catalyzes the oxidation of ammonia to hydroxylamine, which is subsequently oxidized to nitrite (8, 9). Therefore, in order to investigate the roles that microorganisms play in mediating various biogeochemical cyclings and ecosystem functions, a critical issue is to recover the corresponding functional genes in the ecosystem.

Over the past two decades, high-throughput profiling technologies have rapidly evolved and been routinely used to investigate the composition and functional potential of microbial communities in complex ecosystems (10, 11). Multiple major approaches have been developed, including high-density functional gene arrays (e.g., GeoChip), targeted amplicon sequencing of a specific marker gene, and shotgun metagenomic sequencing of the total DNA (12). Of these, functional gene arrays capture the microbial profile by hybridizing environmental DNA with probes designed from known sequences and are therefore termed as a closed format technology (12, 13). High-throughput sequencing approaches, both amplicon and shotgun metagenomic sequencing, hold the potential to discover new sequence variants of the targeted genes. Although these approaches differ greatly, they have been widely used to investigate microbial-mediated biogeochemical processes and ecosystem functions in the past. For instance, they provide insights into the taxonomic and phylogenetic composition, revealing the functional characteristics and potential of entire microbial communities across various environments, including the oceans (14, 15), the human microbiome (16, 17), and the Earth’s microbiome (18).

Conventionally, amplicon sequencing of functional genes is used for community-level investigations of the targeted functional assemblages, whereas shotgun metagenomic sequencing holds the advantage to resolve the relative abundance changes of the targeted functional gene. Such different applications are mainly caused by technical differences in these approaches. Specifically, amplicon sequencing usually captures more sequence variants than shotgun metagenomes for the specific targeted region but fails to quantify the relative abundance of targeted genes as shotgun metagenomes do, due to PCR amplification biases and the inability to provide a comprehensive view of the entire microbial community (19–21). For instance, the commonly used bacterial *amoA* primer set (1F/2R) has a coverage of as low as 29.61% (22). In contrast, shotgun metagenomic sequencing may suffer from information loss due to off-target sequencing, as it usually captures a large proportion of non-target genes, leading to fewer target genes being recovered (23). As a consequence, these different approaches are usually applied in complementary manners (20, 24). Notably, a treasure that is always not mined by many studies is the genetic and phylogenetic information carried by the sequence variants of functional genes, which may provide unique insights into the ecology and evolution of the targeted functional genes. This is especially true for full-length genes, operons, and gene clusters. To gain such necessary information, efficient recovery of full-length targeted functional genes and even operons is desired.

Previously, efforts have been made to recover microbial 16S rRNA and functional genes from shotgun metagenomes through targeted assembly, i.e., assembly of extracted reads for specific genes (25–28). With recent progress in metagenomic data analyses, we expected that this procedure can be performed more efficiently with substantially reduced consumption in computational resource and running time. In this study, using the *amo* gene family as an example, we aimed to recover *amo* genes from shotgun metagenomes via a targeted assembly approach focusing on a specific gene family. The *amo* gene family encodes ammonia monooxygenases that are primarily found in ammonia-oxidizing bacteria (AOB) and ammonia-oxidizing archaea (AOA), playing a crucial role in the nitrogen cycle by converting ammonia to hydroxylamine, which is further oxidized to nitrite. We compared the performance of targeted assembly with conventional full assembly approaches, showing that targeted assembly dramatically outperformed full assembly in terms of computational resource and running time. More importantly, we noticed that targeted assembly recovered higher genetic diversity and more operons than full assembly, bringing more information for ecological and evolutionary investigations. Meanwhile, we also show that assembly-based approaches recovered much higher taxonomic and phylogenetic diversity for the targeted gene than amplicon sequencing approach. This study demonstrates a simple but effective approach to recover ecologically important microbial functional genes in shotgun metagenomes and promotes linking community-level and nucleotide-level biodiversity in microbial ecological studies.

## MATERIALS AND METHODS

### Location description and sample collection

The mudflat intertidal samples used for analyses in this study were collected from a typical mudflat intertidal zone (120.75°E, 36.46°N) located in Qingdao, China, in June 2021. To survey the background pools of regional microbial diversity, a nested sampling scheme was designed. In this sampling scheme, multiple sampling areas were set with different radii (1, 5, 10, 20, 50, 100, and 200 m) from a central point. For each increased sampling area, four samples were collected. A total of 25 sediment samples were collected at the low tide when the sediment was exposed to the air. For each sampling spot, five surface soil cores (approximate depth of 15 cm) were collected and homogenized. The collected sediment samples were immediately transported to the laboratory on ice. Approximately 200 g of sediment was retained for each sample, of which 100 g was temporally stored at 4°C until the analysis of physicochemical properties, while the other 100 g was stored at −80°C for the extraction of total DNA.

### DNA extraction and sequencing

Using the DNeasy PowerSoil Kit (Qiagen, Hilden, Germany), total DNA was extracted from 0.5 g of sediment sample, following the manufacturer’s instructions. DNA quality was assessed by 260/280 and 260/230 nm ratios using a NanoDrop ONE Spectrophotometer (NanoDrop Technologies Inc., Wilmington, DE), and high-quality DNA was stored at −80°C. For amplification of the *amoA* gene of ammonia-oxidizing bacteria (AOB), the primer pair 1F/2R was used (1F, 5′-GGGGTTTCTACTGGTGGT-3′; 2R, 5’- CCCCTCKGSAAAGCCTTCTTC-3′; K = G or T and S = G or C) (29, 30). The amplicon sequencing and the shotgun metagenomic sequencing were conducted on the Illumina HiSeq 2500 platform (Illumina, Inc., San Diego, CA, USA) by Magigene Biotechnology Co., Ltd. (Guangzhou, China).

### Amplicon sequencing processing and analyses

Raw ammonia-oxidizing bacteria (AOB) *amoA* amplicon sequences were processed using the DADA2 pipeline (v1.34.0) (31), which is designed to resolve precise biological sequences from Illumina sequence data and does not involve sequence clustering. Instead of removing primers by length, the “removePrimers” command in DADA2 was used to identify and remove PCR primers from both the forward and reverse reads, allowing for two mismatches. The unpaired reads that remained after primer removal were excluded before further processing by the DADA2 pipeline. The processes, including quality filtering, sample inference, merging of paired reads, deduplication, and chimera identification, were carried out using the default parameters. The functional gene database NCycDB (5) was employed to distinguish high-quality *amoA* genes from *pmoA* genes using the DIAMOND program (v2.1.12) (option: -k 1 -e 0.00001 -id 0.3). Taxonomic assignment for the *amoA* ASVs was carried out by MEGAN6 (32).

### Shotgun metagenome processing, full assembly, and targeted assembly

Raw reads were first trimmed using Trimmomatic (33). The PEAR (v0.9.11) software was used to merge raw Illumina paired-end reads (34), followed by assembly of the merged paired-end and single-end sequences of the metagenomic data sets using MEGAHIT (v1.2.9) (35). Three types of metagenomic assembly approaches were employed and compared, including single-sample assembly, multi-sample assembly, and targeted assembly. For both single-sample and multi-sample assemblies, all reads were used as input to MEGAHIT. In addition, two existing targeted assembly tools, Xander (25) and SAT-assembler (28), were employed. The resulting contigs were compared against NCycDB (5) using DIAMOND (option: -k 1 -e 1 -id 0.3) to identify targeted contigs. For targeted assembly, the paired-end reads were first searched against NCycDB (5) using DIAMOND with the same relaxed parameters (option: -k 1 -e 1 -id 0.3). This relaxed cutoff was chosen to capture a broader range of potentially relevant sequences, ensuring the inclusion of all reads associated with *amo* and *pmo* genes. And the reads with the best hit to *amo* and *pmo* genes were then extracted as input into MEGAHIT and SPAdes (v4.2.0). All extracted reads were used as input for targeted assembly, and no abundance cutoff was employed. For all three assembly approaches, a range of kmer sizes (-k-min 29 -k-max 141) was utilized. For each assembly, the resulting consensus contig data set, generated by merging data from various k-mer sizes, was preserved for subsequent analysis.

The targeted contigs then underwent redundancy removal using CD-HIT (v4.6.2) (36). FragGeneScan (v1.18) was used to predict genes in contigs (options: -complete = 1 -train = complete) (37). Two methods of the USEARCH (v11) program including uchime_ref (38) and uchime_denovo (39, 40) were used to detect potential chimeras using default parameters. In addition, two methods implemented in Mothur (v2.20) (41, 42), namely chimera.perseus (43) and chimera.vsearch (41), were also applied with default settings. The *amo* sequences in NCycDB were used as the database to judge chimeric sequences when using the uchime_ref and chimera.vsearch algorithm. The predicted coding sequences were also searched against the NCycDB functional gene database using the DIAMOND program for functional gene annotation of *amo* and *pmo* genes, with a more stringent threshold (option: -k 1 -e 0.00001 -id 0.3) applied to ensure higher specificity in identifying relevant genes. The contigs containing *amo* genes were then identified and reserved for further analyses.

### Statistical analyses

MetaQUAST (v5.2.0) was used to assess the assembly quality of the three assembly methods, including parameters such as N50, N90, L50, and L90 (44). The EggNOG Database (45–47) and NCBI Database (48, 49) were used for *amo* operon functional annotation. Taxonomic assignments were carried out by the Kraken2 (v2.1.3) program (31). Bowtie2 (v2.5.4) was used to align the raw reads against the contigs (50). Samtools was used to sort and convert SAM files to BAM format (51). The R package “vegan” (v2.7-1) was used to calculate both alpha and beta diversity (52). The spatial turnover rates for DDR were calculated as the linear least-squares regression relationships between the log-transformed geographic distance and community similarity [based on 1 – (dissimilarity of the Bray-Curtis distance metric)]. The slope coefficients for TAR were estimated using linear regression in a log-transformed space based on the observed community richness. DDR and TAR were analyzed separately for bacterial *amo* and archaeal *amo* genes. MAFFT (v7.525) was used for sequence alignment of the extracted genes (53). The aligned sequences were used to infer phylogenetic relationship using FastTree (v2.2) (54). The tree files were uploaded to the Interactive Tree of Life (https://itol.embl.de) for visualization (55).

## RESULTS

### An overview of metagenomic recovery of microbial functional genes

Two major approaches, including amplicon and shotgun metagenome sequencing (56, 57), are commonly used to recover important microbial functional genes in complex environments (Table 1). In general, amplicon sequencing recovers more functional gene variants (e.g., OTUs and ASVs) than shotgun metagenomes, but it faces a critical issue: the recovered genetic diversity is highly constrained by the coverage of PCR primers, which is usually low for functional genes (58). In contrast, shotgun metagenome sequencing holds the potential to recover untargeted genes by the primers but is usually low in the number and abundance of recovered genes via typical assembly approaches (23). In addition, critical issues exist in recovering microbial functional genes via assembly approaches. First, assembly of full metagenome data sets is extremely resource- and time-consuming (25, 26), constraining efficient recovery of targeted functional genes from shotgun metagenomes. Second, shotgun metagenome assembly is likely biased to generate contigs for abundant microbial functional genes (59, 60) but not low abundant ones. Therefore, there is an urgent need to recover microbial functional genes covering high genetic diversity.

**TABLE 1:** An overview of different approaches for recovering microbial functional genes in complex environments

Method	Characteristic	Limitation	Resource consumption	Running time
Amplicon sequencing (61, 62)	Amplicon sequencing targets specific conserved DNA regions by amplifying and sequencing PCR products. Usually, a large number of sequence variants can be recovered.	Amplicon sequencing is highly constrained by the coverage of PCR primers for functional genes, which usually vary dramatically and affect the representativeness of the recovered sequences. Some genes do not even have universal primers available. Full-length sequences are usually not available.	Low	Low
Single-sample assembly (21, 23)	Single-sample assembly recovers targeted functional genes by assembling the reads in each sample to form long contigs. It has the potential to recover full-length sequences for more informative analyses.	The less abundant microbial functional genes can hardly be recovered via single-sample assembly, due to the low coverage of these genes.	Moderate	Moderate
Multi-sample assembly (21, 23)	Multi-sample assembly recovers targeted functional genes by assembling the reads in multiple samples (usually ecological replicates from the same sampling sites) to form long contigs. It has higher potential than single-sample assembly to recover full-length sequences for more informative analyses.	Multi-sample assembly usually costs much more computational resources and running time than single-sample assembly. The requirement of large RAM challenges many microbial ecology laboratories.	High	High
Targeted assembly (25, 26)	Following extracting the reads belonging to the gene of interest, targeted assembly assembles them into long contigs at dramatically declined costs in computational resources and running time.	Only the genes of interest are expected to be recovered and may miss other information carried by full assembly.	Low	Low

**Fig 1:**
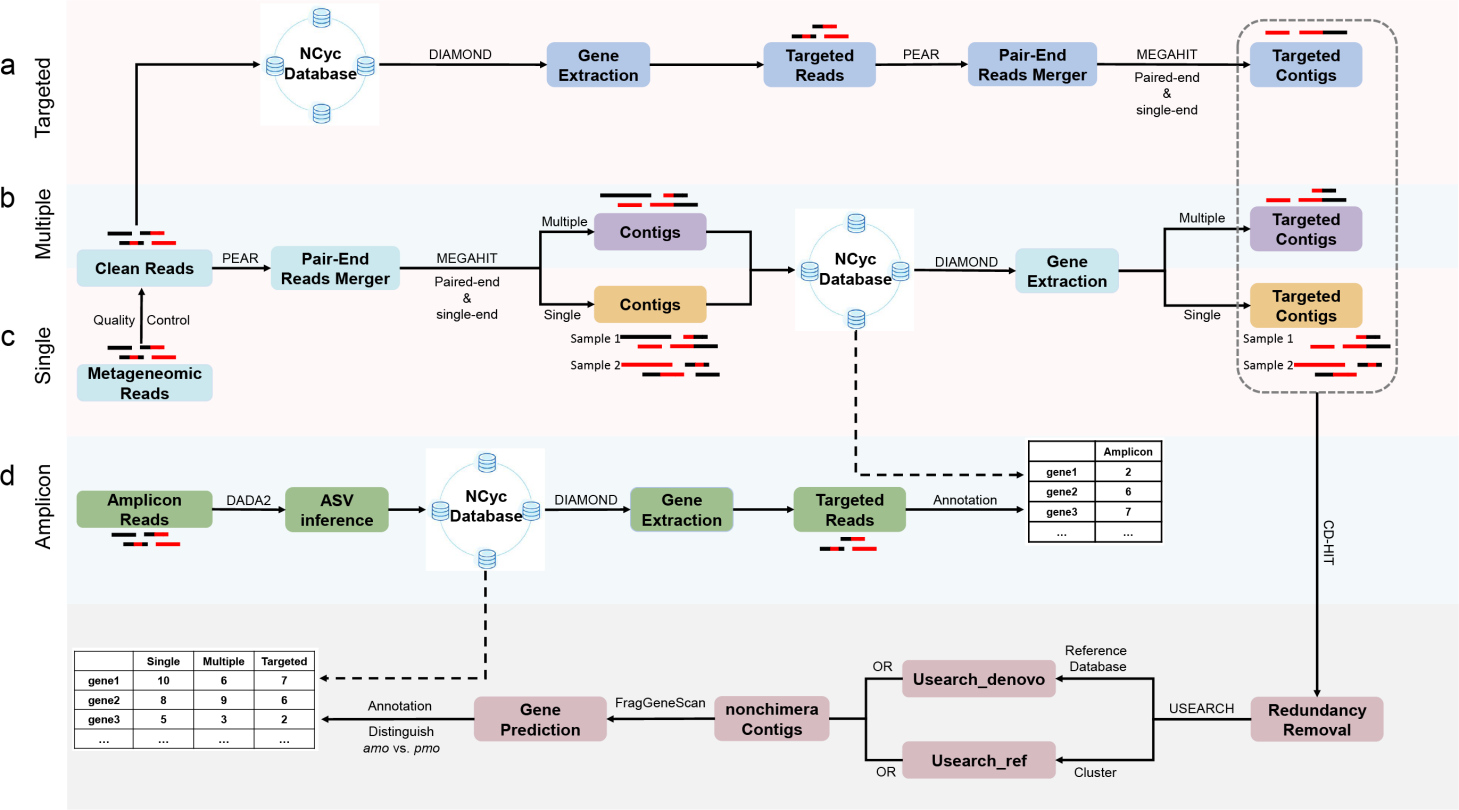
Flowchart of different metagenomic recovery approaches for microbial functional genes. (**a**) Targeted assembly. Using *amo* genes as an example, metagenomic reads were first searched against NCycDB using DIAMOND at relaxed cutoff (option: -k 1 -e 1 -id 0.3), and the reads with best hit to *amo* and *pmo* genes were extracted and subsequently co-assembled using MEGAHIT. (**b**) Multi-sample assembly. Reads of all samples were used as input for MEGAHIT. The resulting contigs were compared against NCycDB using DIAMOND at relaxed cutoff (option: -k 1 -e 1 -id 0.3) to identify potential targeted contigs. (**c**) Single-sample assembly. Similar to multi-sample assembly, the metagenomic reads of each sample were input separately to MEGAHIT. (**d**) Amplicon sequencing. To see how the shotgun metagenome-recovered *amo* genes differ from amplicon sequencing, raw ammonia-oxidizing bacteria (AOB) *amoA* amplicon sequences were analyzed and compared with shotgun *amoA* profiles.

To overcome the abovementioned issues, we tested to recover microbial functional genes via targeted assembly (Fig. 1a) and compared it with other approaches (Fig. 1b through d). Conventionally, shotgun metagenomes are subjected to full assembly, either single-sample or multi-sample, to generate contigs. Gene prediction was then carried out for the contigs. The predicted genes were then searched against orthologous databases to identify the targeted genes, i.e., the *amo* gene family in the current study. Here, the curated functional gene database NCycDB was used for functional annotation and to distinguish *amo* from *pmo* genes. In the targeted assembly approach, the shotgun metagenomic reads were first searched against NCycDB with relaxed parameters. The reads mapped to the targeted genes were then extracted and assembled into longer sequences. Similarly, gene prediction and verification were carried out via NCycDB. The extracted *amo* genes were then subjected to further analyses. In this study, the recovered bacterial *amo* genes via shotgun metagenomes were also compared with an amplicon sequencing data set, aiming to gain insights into how these different approaches may differ in recovering targeted functional genes.

### Performances of different approaches in recovering *amo* genes

**Fig 2:**
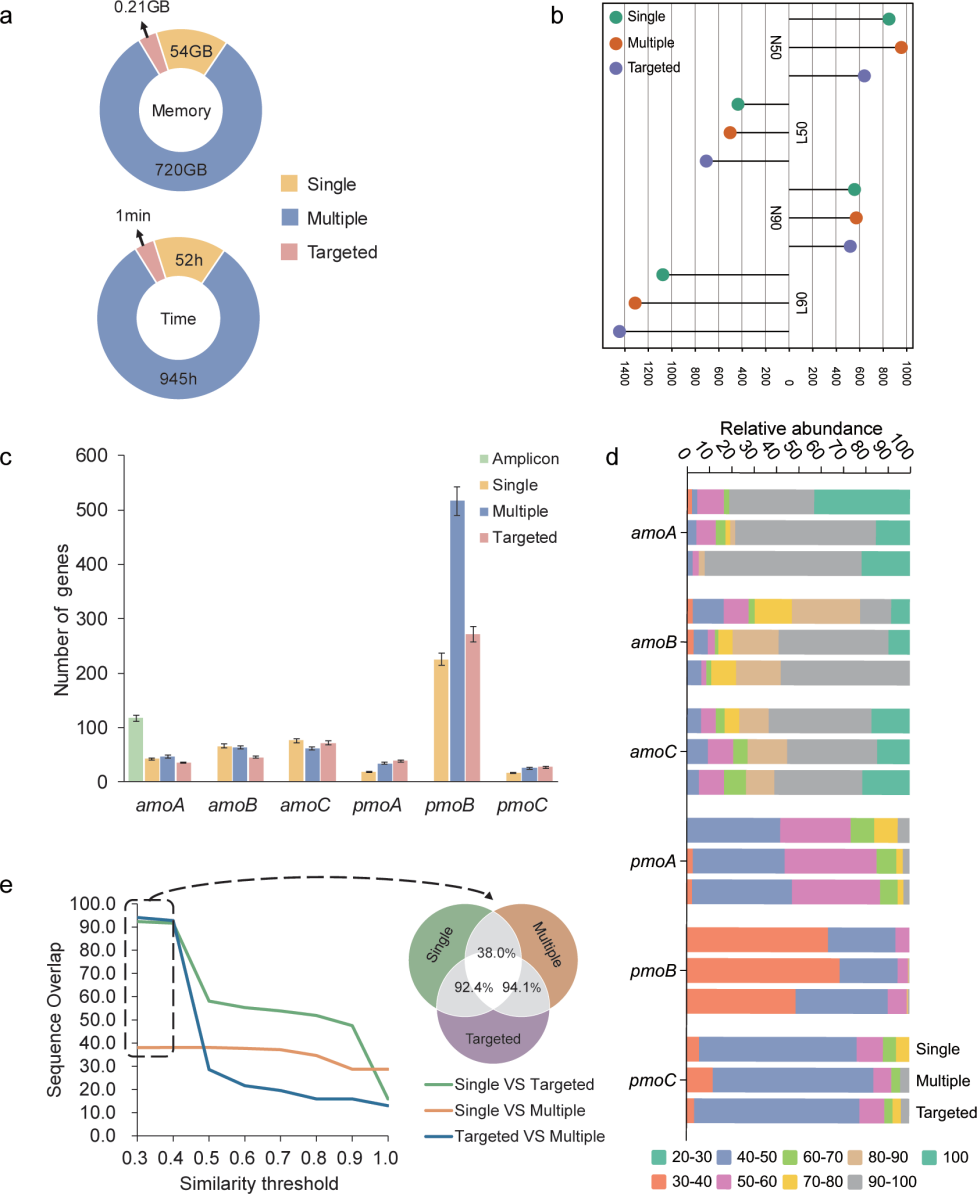
Performances of different approaches in recovering functional genes. (**a**) The consumption of memory and runtime for different approaches. (**b**) Various metrics for assessing assembly quality, including N50, L50, N90, and L90. (**c**) The number of functional genes obtained by different data processing methods. (**d**) Sequence identity of recovered genes by comparing against the NCycDB using DIAMOND (-k 1, -e 0.00001). (**e**) Overlap among the three assembly methods in recovering targeted functional genes. The Venn diagram illustrates the overlap when the similarity identity threshold is 0.3.

We first evaluated the computational efficiency of different assembly approaches in recovering targeted functional genes, focusing on the two most important factors, i.e., the cost of memory and running time (Fig. 2a). As expected, multi-sample assembly required the most computational resource and running time, occupying 720 GB memory and taking 945 running hours for the data set containing 25 samples and 3.57 billion reads. For single-sample assembly, each sample occupied about 54 GB memory and 52 running hours on average, not accounting for the time consumption to predict and extract targeted genes from contigs. In contrast, targeted assembly of extracted reads from all samples consumed only 0.21 GB memory and cost one minute, plus 25 hours for extracting potential *amo* reads against NCycDB using DIAMOND. Such results demonstrated that targeted assembly greatly reduced computational resources and running time compared to conventional approaches, enabling complex metagenomic recovery of targeted gene families on personal computers or even laptops.

Second, we evaluated the assembly quality of these different approaches based on multiple metrics, including N50, L50, N90, and L90 (Fig. 2b). Among these, N50 and N90, respectively, defined as the length at which 50% and 90% of the assembled contigs/scaffolds are of that length or longer, are metrics used to assess assembly continuity. Higher values of N50 and N90 indicate better continuity and longer contigs. L50 and L90 reflect assembly concentrations by indicating the number of contigs/scaffolds that covers 50% and 90% of the assembly length, respectively. Lower values of L50 and L90 denote more concentrated assemblies. Here, the results demonstrated that different assembly methods varied in their performances in terms of these metrics when recovering the targeted *amo* genes. For N90 and L90, the difference between targeted assembly and single-sample or multi-sample assembly was minimal, suggesting comparable quality of functional genes recovered by targeted assembly and single-sample or multi-sample assembly.

Third, we investigated the biological signatures of *amo* genes recovered by these approaches, emphasizing properties, such as gene counts, recovery accuracy, and sequence overlap across different assembly methods. The recovered sequences were searched against NCycDB to distinguish *amo* and *pmo* gene families. As a result, the number of recovered targeted functional genes varied for different assembly approaches, especially for *pmoB* genes. For example, the single-sample assembly recovered 42 *amoA* genes, whereas the multi-sample assembly recovered 46, and the targeted assembly identified 35. And the number of recovered *amoC* genes were 76, 62, and 71, respectively (Fig. 2c). Specifically, we further examined the detection rates of *amoA* genes affiliated with ammonia-oxidizing bacteria (AOB) across amplicon sequencing, single-sample assembly, multi-sample assembly, and targeted assembly, which recovered 117, 8, 15, and 15 sequences, respectively (Fig. S1). We also applied SPAdes for targeted assembly to compare its performance with MEGAHIT. The number of recovered genes was overall comparable between the two assemblers, except for *pmoB*, where SPAdes recovered noticeably more sequences (Fig. S2). In addition, two previously developed targeted assemblers, Xander and SAT-Assembler, were also comparatively tested. Notably, the comparison between these different approaches shall not be over-attended, as the previous methods mainly focus on individual genes, while the current one is extendable to operons/clusters but has the same idea to recover targeted genes with reduced resources and running time. Since the reference marker gene set provided by Xander only includes *amoA* sequences, we limited the comparative analysis to *amoA* genes. Specifically, Xander yielded 18 *amoA* sequences in total, among which 10 were assigned to ammonia-oxidizing archaea (AOA) and eight to ammonia-oxidizing bacteria (AOB) (Fig. S3). Likewise, SAT-Assembler recovered 15 *amoA* (5 AOA, 10 AOB) and 10 *pmoA* sequences (Fig. S4). Furthermore, the recovered sequences were searched against NCycDB to evaluate accuracy. High proportions of assembled genes with high sequence similarity were observed in the targeted assembly, demonstrating a closer match to reference sequences (Fig. 2d). At an identity threshold of 0.3, the mapped ratio of recovered sequences for the targeted genes between single-sample and multi-sample assembly was as low as 38.0%, likely due to the differences in assembly strategies. In contrast, the ratio between targeted assembly and single-sample assembly, as well as that of targeted assembly and multi-sample assembly, was notably high, at 92.4% and 94.1%, respectively (Fig. 2e). These findings suggest that targeted assembly not only exhibited comparable performance in recovering targeted functional gene counts to conventional approaches but also provided greater precision, with a high overlap in the resulting gene sequences.

### Targeted assembly had fewer chimeric sequences than full assembly

**Fig 3:**
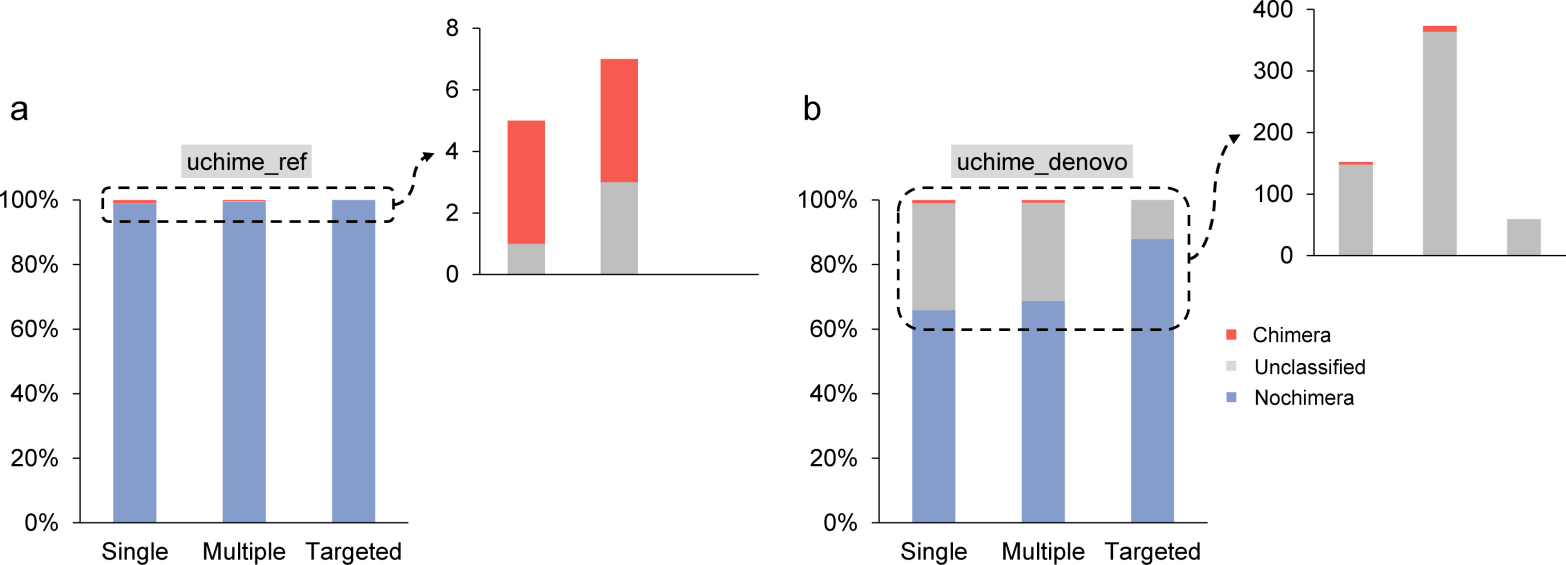
Situation of chimeric sequences of *amo* and *pmo* gene families. Two algorithms for detecting chimeras were used here, including uchime_ref (**a**) and uchime_denovo (**b**).

In addition to performances, another potential issue associated with metagenomic assembly of functional genes is the generation of chimeras. Although chimeras were rarely attended in shotgun metagenomes due to the absence of PCR amplification, the assembly of highly similar sequences may also generate chimera-like sequences. Here, multiple chimera detection algorithms were used to detect potential chimeric sequences. The query is predicted to be chimeric if the score of its alignment to the model exceeds a threshold, or classified as unknown if the uncertainty is high. The reference database is provided by the user (uchime_ref and chimera.vsearch) or constructed *de novo* from the provided sequences (uchime_denovo and chimera.perseus). As a result, when using the uchime_ref mode, both single-sample and multi-sample assembly contained four chimeric sequences and one and three unclassified sequences. In contrast, the targeted assembly produced only non-chimeric sequences (Fig. 3a). When the uchime_denovo mode was used, the number of chimeric sequences detected in the single-sample assembly, multi-sample assembly, and targeted assembly were 4, 9, and 0, respectively. The single-sample and multi-sample assemblies were identified with relatively high proportions of unclassifiable sequences, at 33.3% and 30.6%, respectively, while targeted assembly resulted in a much lower percentage of 12.1%. The proportions of non-chimeric sequences in single-sample assembly, multi-sample assembly, and targeted assembly were respectively 65.8%, 68.7%, and 87.9%, respectively (Fig. 3b). When using chimera.vsearch, five chimeric sequences (0.12%) were detected in the single-sample assembly, two (0.05%) in the multi-sample assembly, and none in the targeted assembly. Accordingly, the proportions of non-chimeric sequences were 99.88%, 99.95%, and 100%, respectively (Fig. S5a). When chimera.perseus was used, no chimeric sequences were detected in any of the three assembly approaches (Fig. S5b). These findings suggested that targeted assembly reduced the likelihood of misassembled reads from different sources.

### More *amo* operons were recovered by targeted assembly

**Fig 4:**
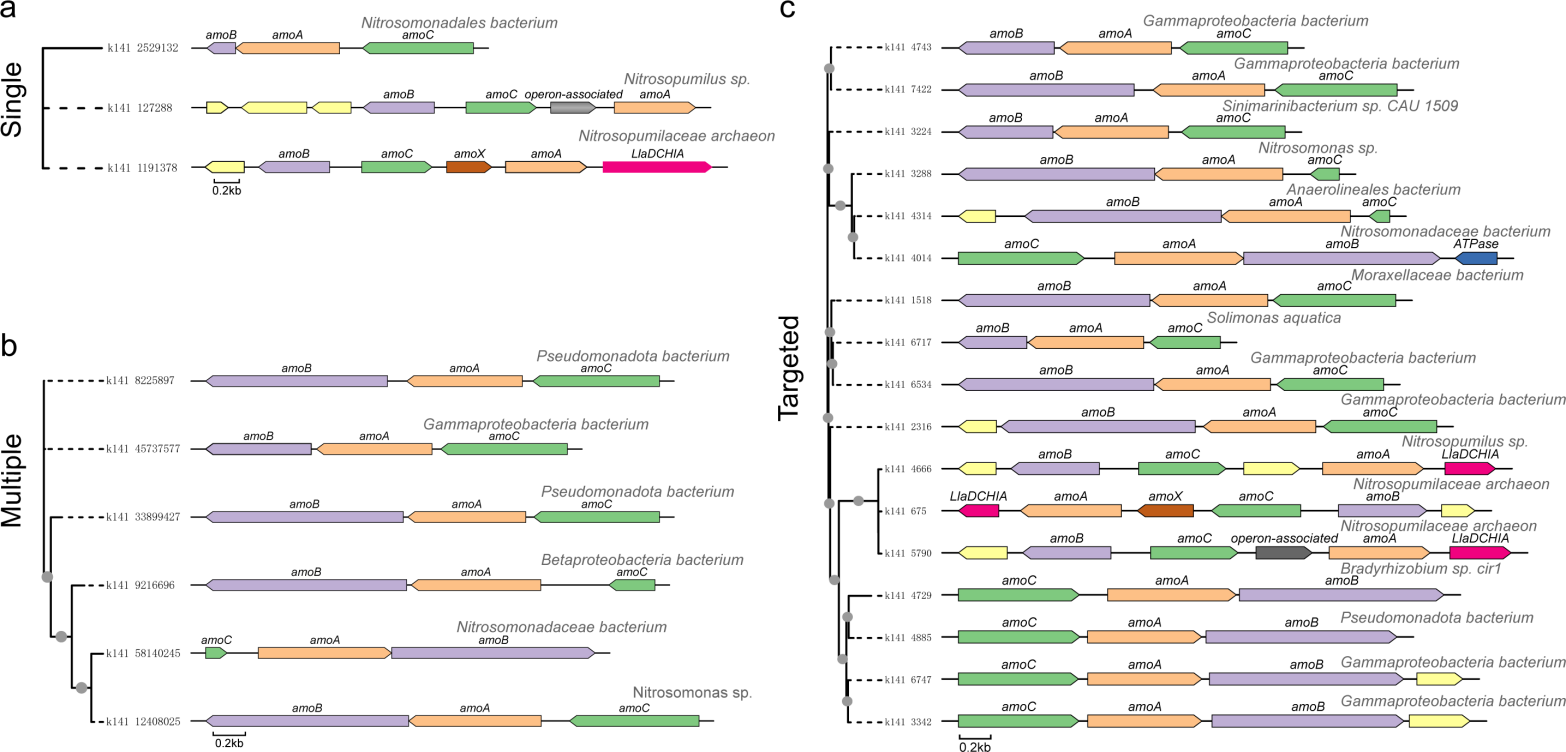
Recovered *amo* operons by different assembly approaches, including single-sample assembly (**a**), multi-sample assembly (**b**), and targeted assembly (**c**). For each analysis, the phylogenetic tree is displayed on the left, while the structures of the *amo* operon of the corresponding sequence are shown on the right. Only sequences containing a complete *amo* operon are presented. Gray dots indicate bootstrap support values greater than 75%.

The *amo* operon is composed of multiple subunits, including the well-recognized *amoC*, *amoA*, and *amoB*. The translational products of these subunits together form the complex ammonia oxidase, which plays a key role in oxidizing ammonia to nitrite and is one of the key enzyme systems in the ammonia oxidization process (63–67). In complex environments, *amo* operons are expected to provide more information than the commonly targeted *amoA* subunits to understand the ecology and evolutionary relationship of ammonia-oxidizing microorganisms. Here, effort was made to screen contigs containing the complete set of *amoA*, *amoB*, and *amoC* subunits (Fig. 4). The results showed that targeted assembly yielded the most complete *amo* operons, with a total of 17 (Fig. 4c), where the yielded numbers for single-sample assembly and multi-sample assembly were 3 and 6, respectively (Fig. 4a and b). Encouraged by the identification of *amoX* and *amoE*, we further incorporated additional subunits (*amoD*, *amoE*, and *amoX*) into the reference database to examine the occurrence of more complete *amo* operons. Notably, three operons containing the full set of *amoABCX* genes were successfully recovered from the targeted assembly data set (Fig. S6). Previous studies have identified multiple new subunits, such as *amoXYZ*, in addition to the known *amoCAB* subunits (68, 69). The emergence of the Nitrososphaeraceae family has resulted in the scattering of *amo* subunit gene across the genome except *amoA* and *amoX*, which are normally linked (9). The contig sequences obtained by targeted assembly and single-sample assembly in this study also demonstrated the presence of *amoX* subunit in the *amo* complex (Fig. 4c). In *N. europaea*, two nearly identical *amo* operon copies, composed of *amoC*, *amoA*, and *amoB* (*amoCAB*), are detected in the genome, followed in the downstream by two open reading frames, namely Orf4 and Orf5 (70, 71). Of these, Orf5 is named *amoD* (72), and Orf4 (also known as *amoE*) (70) is described as an exact gene duplication of Orf5, present in all β-AOB (70, 73). Both genes, *amoD* and *amoE*, have highly conserved sequences and are similarly localized in the *amo* operon, suggesting that they may encode proteins that play important roles in ammonia oxidation (74). In this study, the *amoE* gene was also identified by targeted assembly, providing additional clues for the study of ammonia oxidation (Fig. S7).

Genomically, biological processes are frequently encoded by co-transcribed neighbor genes, which are termed operons. In general, operon-associated genes often belong to evolutionary gene families with similar biological functions. In this study, operon-associated genes were also observed in some sequences. Specifically, gene families were rarely recovered before or after the *amo* operons obtained by multi-sample assembly in this study. In contrast, genes such as LlaDCHIA and ATPase were found near or within the *amo* operons recovered by single-sample assembly and especially targeted assembly (Fig. 4c). Integrating the above message, the results suggested that targeted assembly outperformed full assembly in obtaining *amo* operons.

### Targeted assembly recovered high *amo* diversity in mudflat intertides

We then investigated the diversity of *amo* gene families recovered by different approaches from different angles, including the phylogenetic diversity, taxonomic composition, and ecological patterns.

**Fig 5:**
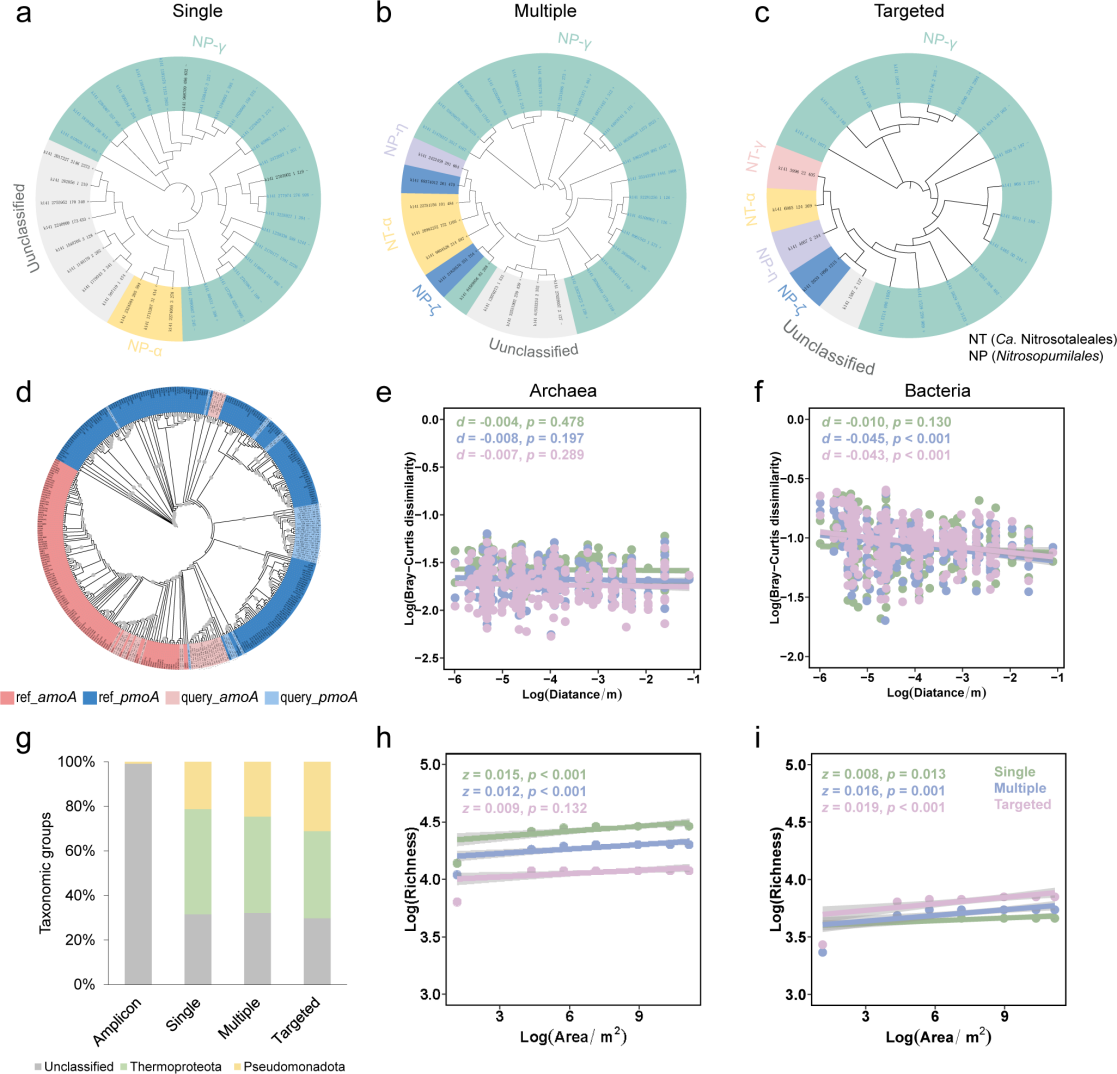
Phylogenetic trees, spatial scaling patterns, and composition of *amo* gene family by different approaches. (**a–c**) Phylogenetic and lineages of archaeal *amoA* gene recovered by different approaches. Different colored label backgrounds represent different taxonomic ranks. Blue labels represent archaea and gray for unclassified. (**d**) Phylogenetic tree of *amoA* and *pmoA* genes recovered by targeted assembly. Gray dots indicate bootstrap support values greater than 75%. (**e–f**) DDR of archaeal and bacterial *amo* gene family. (**g**) Taxonomic composition of *amo* gene family at the phylum level. (**h–i**) TAR of archaeal and bacterial *amo* gene family.

First, we explored the phylogenetic diversity of *amo* genes obtained by different recovery approaches (Fig. 5a through c). In particular, we focused on the lineages of archaeal *amoA* genes. Previous studies have constructed a high-resolution phylogenetic tree of the archaeal *amoA* genes and defined a multi-level archaeal *amoA* taxonomy (75). The *amoA* phylogeny comprises four basal lineages, including NC (*Ca. Nitrosocaldales*), NS (*Nitrososphaerales*), NT (*Ca. Nitrosotaleales*), and NP (*Nitrosopumilales*). Here, by comparing against the previously *amoA* phylogenetic clades (75), we found that the intertidal archaeal *amoA* genes obtained by these methods were categorized as NT (*Ca. Nitrosotaleales*) and NP (*Nitrosopumilales*) lineages and that the number of NP lineage was generally greater than that of NT lineage. Further subclassification revealed that single-sample assembly contained the least number of “super-clades,” including NP-α and NT-γ. Multi-assembly identified four “super-clades,” including NT-α, NP-γ, NP-η, and NP-ζ, whereas targeted assembly identified five, including NT-α, NT-γ, NP-γ, NP-ζ, and NP-η (52). This observation highlights the advantages of targeted metagenomic assembly, especially its ability to discover sequence variants not identified by full assembly.

Additionally, a phylogenetic tree was constructed to further differentiate the *amoA* and *pmoA* genes recovered in this study. Reference sequences for *amoA* and *pmoA* genes were obtained from NCycDB. In the single-sample assembly, the query and reference sequences did not cluster effectively, and the distinction between *amoA* and *pmoA* sequences was unclear, as they were intermixed (Fig. S8). In contrast, both multi-sample and targeted assemblies resulted in more accurate clustering of query and reference sequences. However, a proportion of *amoA* sequences remained misclassified within the *pmoA* clade. Importantly, the targeted assembly yielded fewer misclassified *amoA* sequences than the multi-sample assembly, suggesting that targeted assembly likely provided more accurate recovery of the *amoA* genes and better differentiation between *amoA* and *pmoA* sequences (Fig. 5d; Fig. S8).

Second, we investigated two typical spatial scaling modes, including DDR and TAR. The first spatial scaling pattern that we analyzed was DDR (Fig. 5e and f; Fig. S9a), which describes the pattern in which biomes become more dissimilar in composition with increasing geographic distance. The slope coefficients representing the correlation between the log-transformed geographic distance and the community similarity were calculated for DDR. Significant DDR patterns were observed for bacterial (multi-sample and targeted assembly) but not for archaeal *amo* genes. For archaeal *amo* genes, DDR patterns were observed as follows: single-sample assembly (*d* = −0.004, *P* = 0.478), multi-sample assembly (*d* = −0.008, *P* = 0.197), and targeted assembly (*d* = −0.007, *P* = 0.289). Meanwhile, for bacterial *amo* genes, DDR patterns were as follows: amplicon (*d* = −0.009, *P* = 0.445), single-sample assembly (*d* = −0.010, *P* = 0.130), multi-sample assembly (*d* = −0.045, *P* < 0.001), and targeted assembly (*d* = −0.043, *P* < 0.001). The second spatial scaling pattern we analyzed was TAR (Fig. 5h and i; Fig. S9b), which describes the increase in species richness with increasing sampling area. We calculated the *z*-values representing the slope of the taxa-area relationship in log-log space for different genes and data-processing methods. Significant TAR patterns were observed for both archaeal and bacterial *amo* genes. For archaeal *amo* genes, the TAR patterns were as follows: single-sample assembly (*z* = 0.015, *P* < 0.001), multi-sample assembly (*z* = 0.012, *P* < 0.001), and targeted assembly (*z* = 0.009, *P* = 0.132). For bacterial *amo* genes, the TAR patterns were as follows: amplicon (*z* = 0.109, *P* < 0.001), single-sample assembly (*z* = 0.008, *P* = 0.013), multi-sample assembly (*z* = 0.016, *P* = 0.001), and targeted assembly (*z* = 0.019, *P* < 0.001).

Third, we observed that similar *amo* taxonomic compositions were yielded by different approaches at the phylum level (Fig. 5g). Specifically, for bacteria, *Pseudomonadota* was the sole phylum identified, comprising 0.85% amplicon, 21.2% single-sample assembly, 24.6% of multi-sample assembly, and 31.1% of targeted assembly. For archaea, *Thermoproteota* was the only phylum detected, representing 47.3% of single-sample assembly, 43.3% of multi-sample assembly, and 39.1% of targeted assembly. This suggested a high prevalence of *Thermoproteota* in the analyzed samples, underscoring the ecological importance of archaea in intertidal mudflats. These observations provided valuable insights into the microbial ecology of mudflat intertides and contributed to our understanding of the functional roles of archaea in biogeochemical cycles within these environments.

## DISCUSSION

Due to its highly comprehensive and in-depth probing capability in recovering microbial diversity, functional potential, and ecological characteristics, shotgun metagenomic sequencing has been widely used in the fields of microbial ecology (76) and environmental science (77). Meanwhile, the associated analytical tools are constantly developed and improved to adapt to the needs of complex data analysis, mainly including read-based and assembly-based. Metagenomic assembly plays an essential role in recovering microbial genomic sequences. However, its high cost in computational resources (e.g., CPU and RAM) and running time are equally significant. Although current metagenome assembly tools, such as MEGAHIT (35), SPAdes (78), and SOAPdenovo (79), meet the current needs in many cases, they still suffer from high resource consumption and long computation times. For example, assembling a data set of tens of millions of reads may require tens to hundreds of gigabytes of RAM (e.g., the assembly of 3.57 billion reads using MEGAHIT consumed 720 GB of memory in this study), which is challenging for many microbial ecological researchers. Furthermore, with the ongoing technical advances in high-throughput sequencing, the volume of sequencing data is expanding rapidly, resulting in greater resource requirements. Additionally, the complexity and time cost of the assembly are also influenced by factors, such as the quality and quantity of data, the complexity of community structures, and the efficiency of assembly parameters and algorithms (25, 35).

Currently, read-based analysis becomes easily feasible with the construction of targeted functional gene databases, such as NCycDB (5) and SCycDB (80), and the development of rapid database searching tools, such as DIAMOND (81), BWA (82), and RAPSearch (83). These approaches have the advantage of being fast and less resource-consuming but are less informative when compared to the contigs, such as the missing of synteny information and full-length gene sequences. Therefore, here we aimed to more efficiently recover microbial functional genes of interest through targeted assembly, reducing the required computational resources and gaining more genetic information for the targeted genes. Compared to conventional assembly (single-sample and multi-sample assembly) followed by targeted gene screening, this strategy is expected to save huge amounts of resources and time. Here, the targeted genes were filtered based on relaxed sequence similarity, simplifying processes such as HMM model building and large number of iterative searching (25–27). Notably, the explored targeted assembly here is not limited to existing databases but allows users to use custom databases of interested genes.

Unlike full assembly, the extraction of targeted reads using relaxed parameters may introduce potential false positives in the assembled contigs in the very beginning. Therefore, a second step of filtering non-targeted genes was performed against the NCycDB using more stringent parameters, followed by sophisticated phylogenetic and comparative genomic analysis. Additionally, this study also looked into the potential chimerism issue associated with targeted assembly, which was previously rarely attended in shotgun metagenomes (25–28). Chimeras can result from PCR-induced recombination or misassembly of short reads. Undetected chimeras can be misinterpreted as new sequence variants, leading to inaccurate functional predictions and inflated diversity analyses. Multiple chimera detection methods have been developed in the past, such as Pintail (84), Mallard (85), Bellerophon (86), ChimeraVserch (41), Perseus (43), and UCHIME (40). Here, multiple chimera detection algorithms demonstrated that targeted assembly has a lower chimerism ratio than single- and multi-sample assembly.

Ammonia monooxygenase (AMO) is a three-subunit enzyme (87) expressed by one, two, or three copies of polygenic operons in ammonia-oxidizing autotrophic bacteria and archaea (88–90). Not all ammonia-oxidizing bacteria and archaea have *amo* operons that strictly contain the three subunits. Variations in the *amo* operons have been observed, with some organisms exhibiting operons that lack one or more of these subunits (91). Moreover, it has been recently found that there are some conserved hypothetical proteins associated with *amo* gene clusters, such as *amoX* (69), *amoY*, and *amoZ* (9). In the *amoABCX* cluster, *amoA* and *amoX* are usually linked (9), which was also observed in the study. There are also some genes located downstream of the *amo* operon, such as *amoE* and *amoD* (92). The *amoE* gene was also recovered in the contigs obtained from targeted assembly. Hence, targeted assembly not only obtained more *amo* operons but also recovered more genetic information related to *amo* operon, providing more comprehensive information for studying amo operons in metagenomics. Notably, the maturing long-read sequencing (e.g., PacBio, Nanopore) is expected to offer potential solutions for (mis-)assembly, chimeras, and recovery of operons/gene clusters (93, 94). However, current limitations, such as lower throughput, higher error rate, and higher cost than next-generation sequencing (e.g., Illumina), hinder its wide application in microbial ecology and environmental science.

In summary, using *amo* as an example, this study demonstrated the feasibility to efficiently obtain long microbial functional gene variants from shotgun metagenomes via targeted assembly. Comparing to conventional assembly approaches (e.g., single- and multi-sample assembly), targeted assembly holds the advantage of obtaining high-quality functional profiles and more informative genetic elements, using minimal computational resource and time. The *amo* gene variants recovered by targeted assembly were found with higher phylogenetic diversity and more complete operon structures. This study is expected to provide valuable technical routes for functional gene-targeted studies in shotgun metagenomics, with potential applicability to diverse environmental settings, such as soils and open oceans, and additional testing.

## Data Availability

Raw reads of mudflat intertidal metagenomes generated in this study have been deposited in the NCBI Sequence Read Archive (SRA) database under project ID PRJNA1186844. All custom scripts and selected data generated during the analysis are publicly accessible on GitHub (https://github.com/Mengqi-W/Targeted-Assembly).

## SUPPLEMENTAL MATERIAL

Supplemental figures msystems.00620-25-s0001.pdf Fig. S1 to S9.

ASM does not own the copyrights to Supplemental Material that may be linked to, or accessed through, an article. The authors have granted ASM a non-exclusive, world-wide license to publish the Supplemental Material files. Please contact the corresponding author directly for reuse.
