# Supplementary material for: Targeted assembly recovers high ammonia monooxygenase diversity in mudflat intertides
Source: mSystems. 2025 Sep 24;10(10):e00620-25. doi: 10.1128/msystems.00620-25 (PMC12542656; doi:10.1128/msystems.00620-25)
Supplement: Supplemental figures — Fig. S1 to S9. [file msystems.00620-25-s0001.pdf]

Targeted assembly recovers high ammonia monooxygenase diversity in mudflat intertides

Mengqi Wang[1], Wen Song[1], Jiayin Zhou[1], Mengzhi Ji[1], Kai Ma[1], Yan Li[1], Qichao Tu[1, 2, *]

[1] Institute of Marine Science and Technology, Shandong University, Qingdao, China

[2] Southern Marine Science and Engineering Guangdong Laboratory (Zhuhai), Guangdong, China

[*] Correspondence should be addressed to Qichao Tu, Email: tuqichao@sdu.edu.cn

Running title: Targeted assembly recovers high *amo* genetic diversity

Supplementary Figures

Supplementary Fig 1. The number of AOB-affiliated *amoA* genes obtained by different data processing methods.

Supplementary Fig 2. The number of functional genes recovered by targeted assembly obtained using MEGAHIT and SPADES.

Supplementary Fig 3. The number of *amoA* genes obtained by Xander.

Supplementary Fig 4. The number of functional genes obtained by SAT-assembler.

Supplementary Fig 5. Situation of chimeric sequences of *amo* and *pmo* gene families.

Supplementary Fig 6. Recovered more complete *amo* operons by targeted assembly.

Supplementary Fig 7. All types of *amo* operons identified in this study.

Supplementary Fig 8. Phylogenetic trees of *amoA* and *pmoA* genes recovered by single-sample and multi-sample assembly.

Supplementary Fig 9. Spatial scaling patterns of bacterial *amoA* genes recovered by amplicon sequencing.

Supplementary Figures

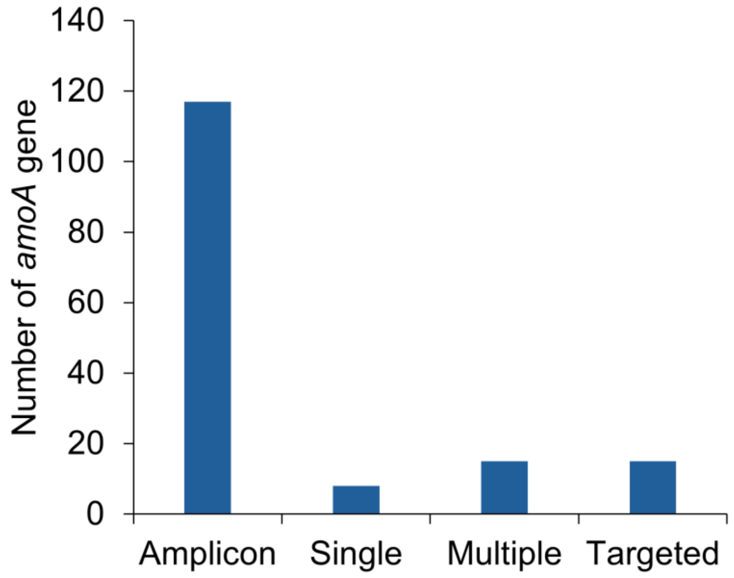

Supplementary Fig 1. The number of AOB-affiliated *amoA* genes obtained by different data processing methods.

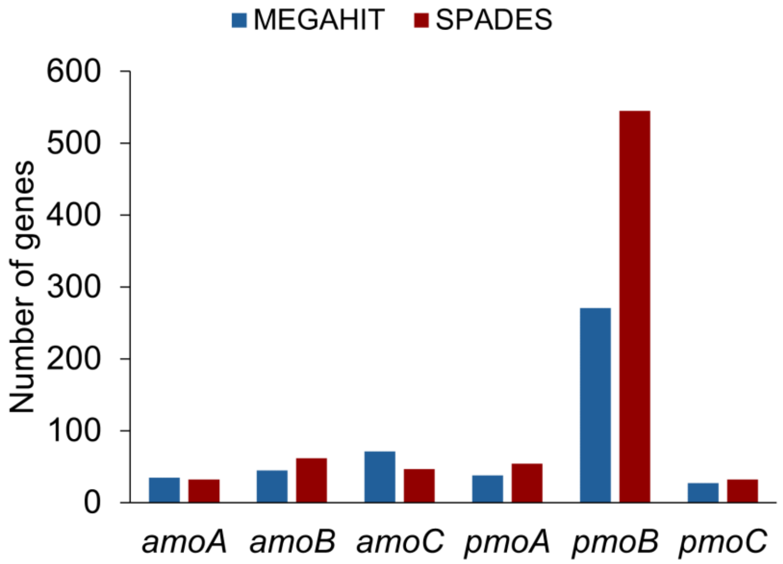

Supplementary Fig 2. The number of functional genes recovered by targeted assembly obtained using MEGAHIT and SPADES.

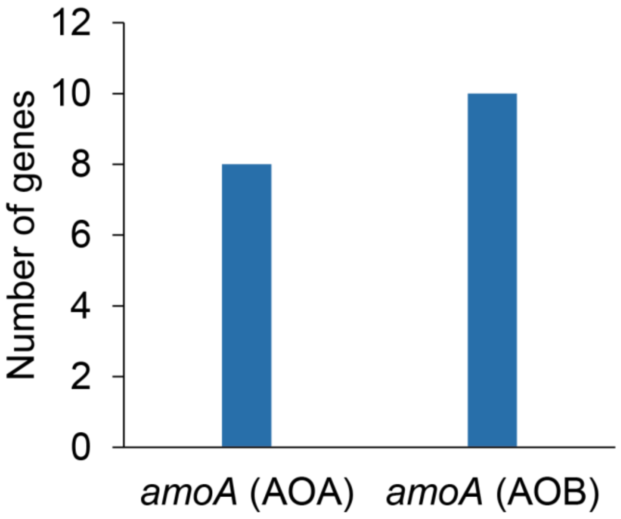

Supplementary Fig 3. The number of *amoA* genes obtained by Xander.

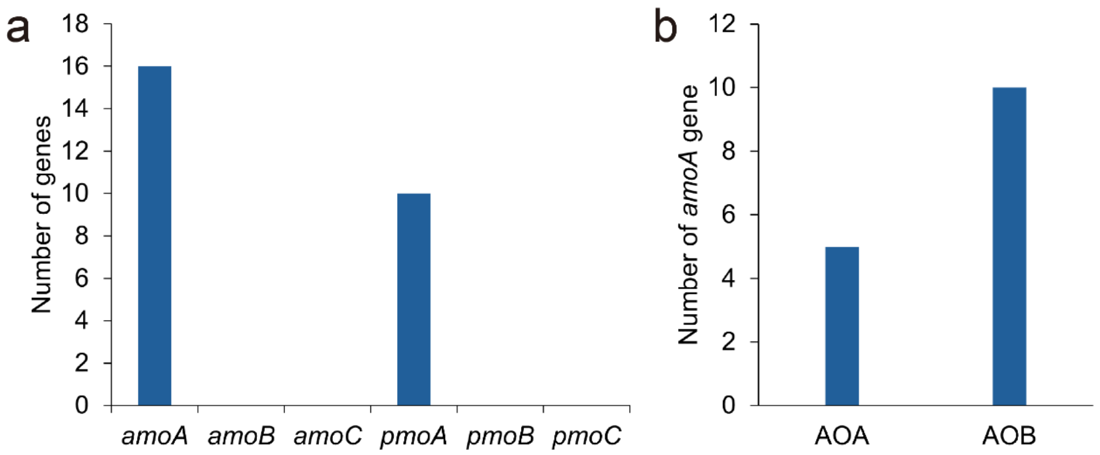

Supplementary Fig 4. The number of functional genes obtained by SAT-assembler (a). The number of *amoA* genes affiliated with ammonia-oxidizing archaea (AOA) and ammonia-oxidizing bacteria (AOB) (b).

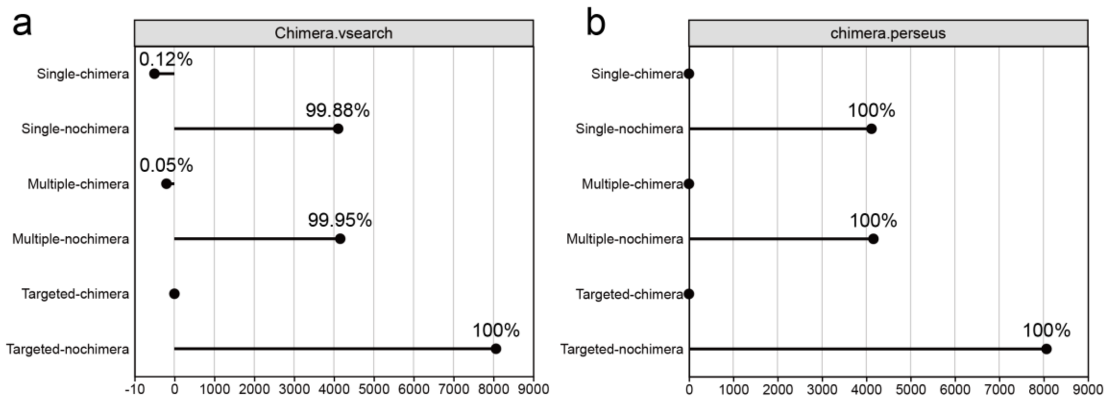

Supplementary Fig 5. Situation of chimeric sequences of *amo* and *pmo* gene families. Two algorithms for detecting chimeras were used here, including chimera.vsearch (a) and chimera.perseus (b), applied to the single-sample assembly, multi-sample assembly, and targeted assembly.

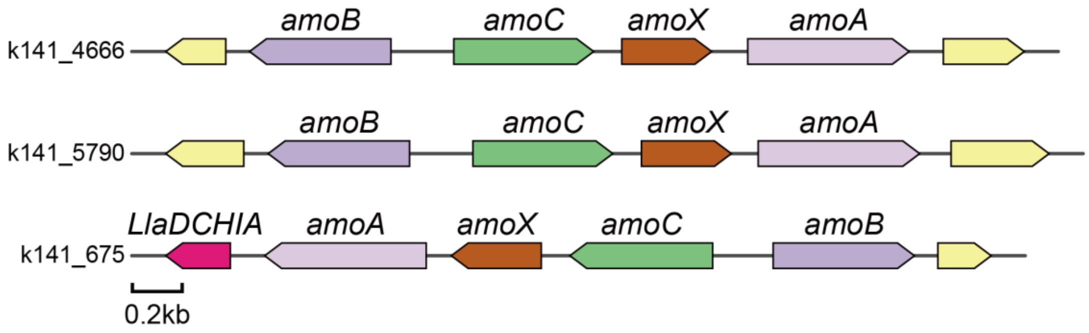

Supplementary Fig 6. Recovered more complete *amo* operons by targeted assembly.

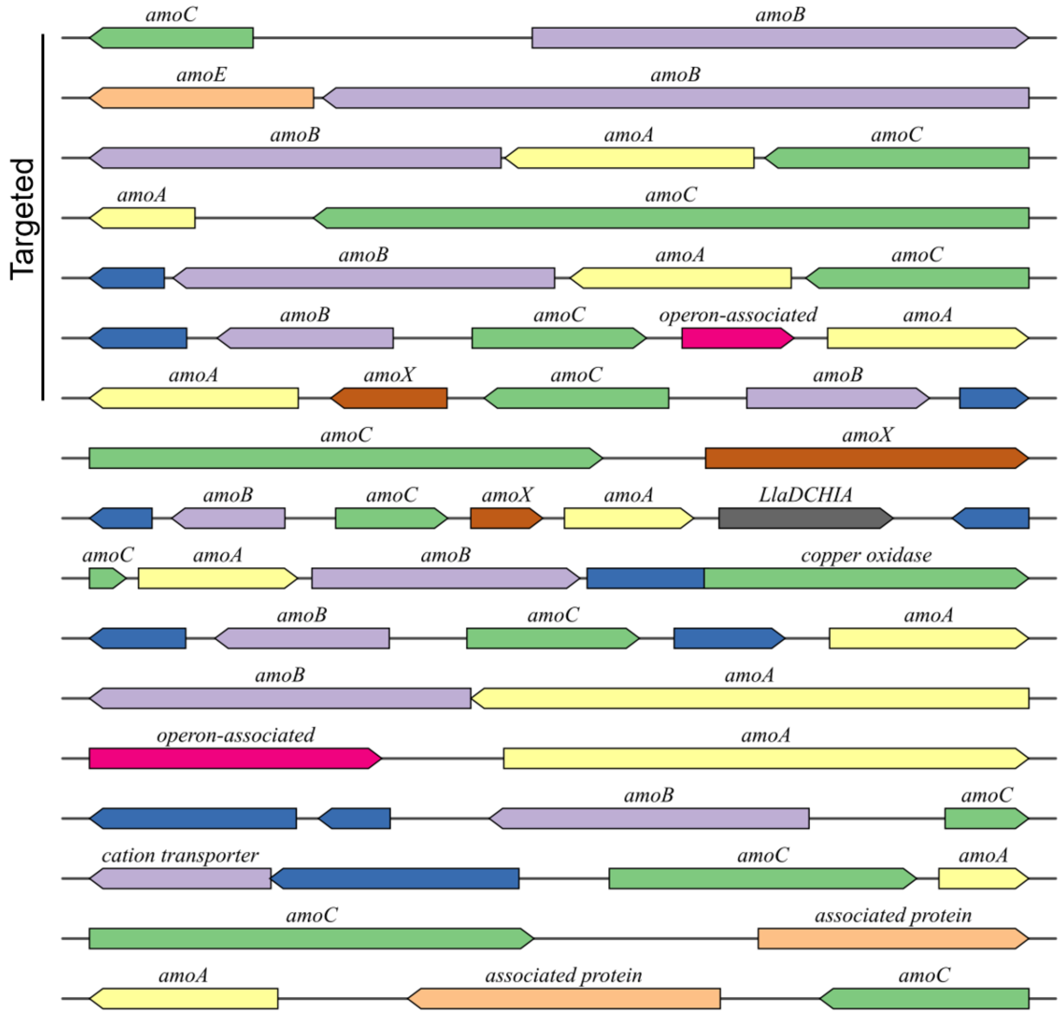

Supplementary Fig 7. All types of *amo* operons identified in this study. The operons containing at least one gene of the *amoCAB* subunits identified by the single-sample assembly, multi-sample assembly, and targeted assembly were included.

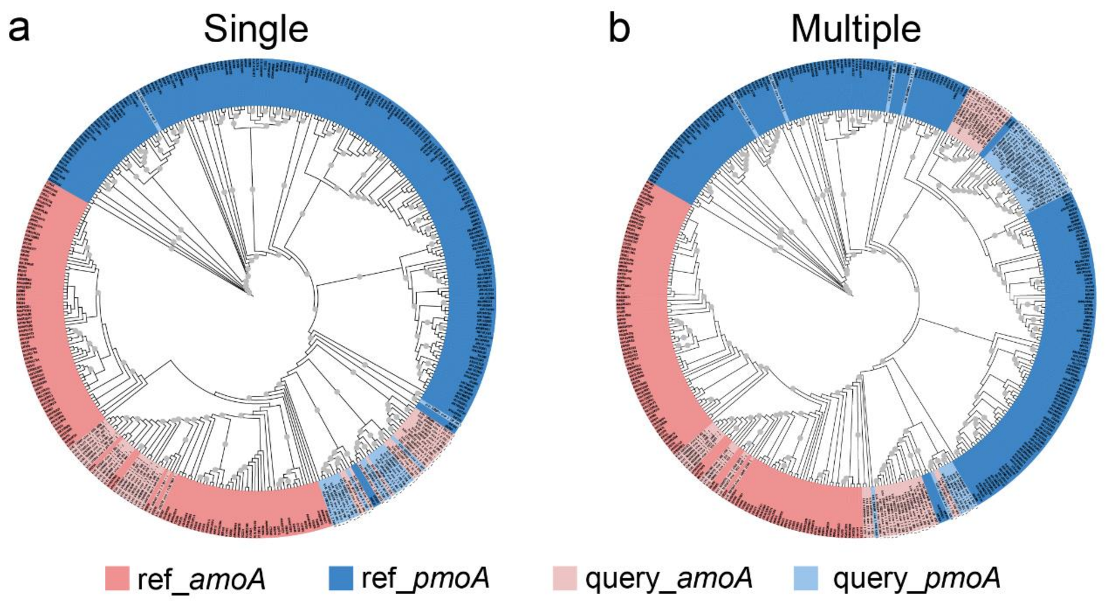

Supplementary Fig 8. Phylogenetic trees of *amoA* and *pmoA* genes recovered by single-sample assembly (a) and multi-sample assembly (b). Gray dots indicate bootstrap support values greater than 75%.

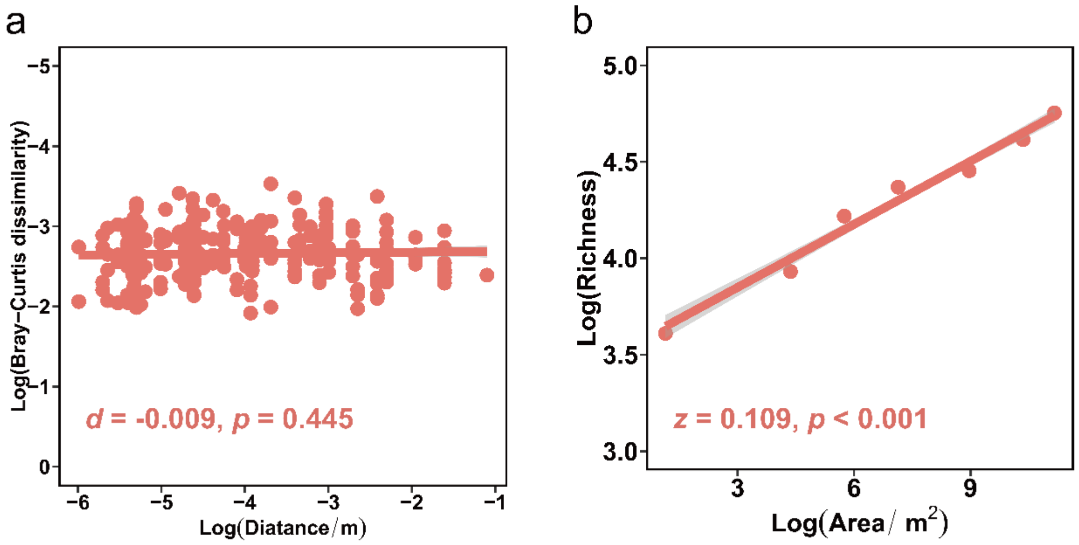

Supplementary Fig 9. Spatial scaling patterns of bacterial *amoA* genes recovered by amplicon sequencing, including DDR (a) and TAR (b).
